# Editorial: Long- and post-COVID syndromes: immune mechanisms and therapeutic strategies

**DOI:** 10.3389/fimmu.2026.1935027

**Published:** 2026-07-24

**Authors:** Luca Roncati, Robert Weissert

**Affiliations:** 1 Department of Life Sciences, Health, and Health Care Professions, School of Medicine and Surgery, Link Campus University, Rome, Italy; 2 Department of Neurology, University of Regensburg, Regensburg, Germany

**Keywords:** angiotensin type-1 receptor, arginine, chronic fatigue syndrome, long-COVID, myalgic encephalomyelitis, NK cells, post-COVID, SARS-CoV-2

Long-term coronavirus disease (long-COVID) is an umbrella term that, according to the UK’s National Institute for Health and Care Excellence, encompasses the debilitating effects of severe acute respiratory syndrome coronavirus 2 (SARS-CoV-2) infection lasting 4 to 12 weeks and 12 weeks or more, not explained by an alternative diagnosis; in the latter case, the term post-COVID syndrome can be adopted as a synonym (1). It is therefore a post-acute infection syndrome sharing similarities with post-Ebola syndrome and others (2). More rarely, it can also occur after the anti-SARS-CoV-2 vaccination (Limongelli et al.), although vaccination usually offers some protection against long-COVID (Guimarães et al.). Risk factors include, but are not limited to, female sex, older age, obesity, smoking (Alshammari et al.), concomitant respiratory diseases (asthma and chronic obstructive pulmonary disease), more severe infection at onset, and mental health problems (3, 4). Common symptoms are represented by fatigue, myalgia, post-exertional malaise or dyspnea, chest pain, palpitations, cognitive and memory impairment (“brain fog”), initial loss of smell and taste, sleep disorders, headaches, depression and anxiety (Escrivá et al.). There is therefore an overlap in symptoms with myalgic encephalomyelitis/chronic fatigue syndrome, and patients report significant impairments in daily life and work ability, accompanied by a high psychological burden (Uecker et al.). As of 2025, the prevalence of long-COVID is estimated to be around 7% in adults and 1% in children having had COVID-19 (5); in practice, over 400 million people worldwide have experienced long-COVID in their lifetime (5). Various mechanisms play a role in the pathogenesis of long-COVID, including organ damage (4), endothelial dysfunction and blood clotting (6), persistent infection or reactivation of latent viruses (7), autoimmunity (4), altered intestinal permeability (Leclerc et al.; Andus et al.), and gut microbiome imbalance (Wang et al.). In particular, our attention has been focused on immune mechanisms and therapeutic strategies.

Ray et al. have identified natural killer cells, expressing the cluster of differentiation 56 (CD56), as key sentinels of long-COVID in terms of quantitative and functional deficits associated with suppression of neurosensory pathways; on the other hand, Kronstein-Wiedemann et al. have detected a parallel increase in CD56-positive monocytes, an expression of a dysregulated monocyte compartment. In their proof-of-concept, Melamed et al. have shown how complement (C) activation due to low levels of C1-esterase inhibitor play a co-role in long-COVID. Moreover, according to Rossi et al., post-COVID syndrome patients have lower levels of anti-spike antibodies compared to COVID-recovered controls, but present an enhanced immunoglobulin G4 switch after the third vaccine dose. Rodriguez-Perez et al. and Vogelgesang et al. have suggested a broad-spectrum autoimmune response targeting serum angiotensin type-1 receptor, neurofilament light chain and/or mitochondrial proteins. Interestingly, Hoheisel et al. have identified autoantibodies directed against Epstein-Barr virus mimicking arginine-rich sequences in human proteins, suggesting a potential role for molecular mimicry in the pathogenesis of long-COVID. All this aligns well with the beneficial effects of arginine supplementation found in long-COVID patients at an oral dose of 1.66 g twice a day for one month (8, 9). Baptista et al. have compiled a list from the scientific literature of fourteen top interventions to treat long-COVID: antihistamines, antivirals, coenzyme Q10, colchicine, exercise training, guanfacine, intravenous immunoglobulins, metformin, monoclonal antibodies, multicomponent rehabilitation packages, naltrexone, nattokinase, nicotine, vagus nerve stimulation. In this regard, Pridgen & Putrino call the need for a large trial of combination antivirals (nirmatrelvir/ritonavir plus valacyclovir), while Scott et al. in their work underline the usefulness of exercise. In addition, the effectiveness of ultramicronized palmitoylethanolamide has recently been described in post-COVID neuropathy (10). Finally, Wang et al. have reviewed the literature also for the usefulness of acupuncture, allowing the knowledge of Eastern medicine to be merged with that of Western medicine in the treatment of this multi-faceted disease, which deserves further research.
